# The prevalence of CHEK2 and CYP1B1 mutations/polymorphisms in urinary bladder cancer

**DOI:** 10.1186/1897-4287-10-S4-A20

**Published:** 2012-12-10

**Authors:** Monika Banaszkiewicz, Maria Constantinou, Michał Pietrusiński, Łukasz Kępczyński, Adam Jędrzejczyk, Marek Rożniecki, Piotr Marks, Bogdan Kałużewski

**Affiliations:** 1 Department of Medical Genetics, Medical University of Łódź, Łódź, Poland; 2 II Clinic of Urology, Medical University of Łódź, Łódź, Poland; 3 John Paul II Regional Hospital, Departament of Urology andl Źlinic of Urology, Bełchatów, Poland; 4 Clinic of Urology - “Lekarze Urolodzy Marek Rożniecki i Partnerzy” NZOZ Łask, Poland

## 

This work was supported by the State Committee for Scientific Research (KBN Poland) grant No. N401 197 32/4212.

## Introduction

Urinary bladder carcinoma ranks the fourth position in malignancy incidence rates in men (6.1%) and the 17th position in women (1.6%). In general, neoplastic diseases, should be approached from two perspectives: prevention with implementation of prophylactic measures and early diagnostics. Prophylactics is possible in the preclinical phase of neoplasm, being both justified and plausible in patients from high-risk groups. Thus, it is particularly important to select such groups, not only by referring to environmental carcinogenic factors (occupational and extra-occupational) but also from genetic predisposition, which may be conductive for neoplasm formation. The mutations / polymorphisms of *CHEK2* and *CYP1B1* genes predispose to neoplasm via multiorgan mechanisms, while the human papilloma virus (HPV) may actively participate in the neoplastic transformation towards urinary bladder carcinoma as an environmental factor.

## Goals of study

The primary goal of the study was an evaluation of the incidence of *CHEK2* gene mutations, *CYP1B1* polymorphism and of the oncogenic types of HPV in a group of patients with urinary bladder carcinoma and in control groups. The secondary goals included a comparison of the incidence of the above-mentioned mutation/polymorphism and of persistent infection with HPV in the studied group of patients and in the control group, with a subsequent determination of the effects, which those factors could have exerted on the neoplastic process development. In general, the studies were undertaken to determine whether there is any genetic and environmental predisposition to urothelial carcinoma and to evaluate possible advantages of the used methods for efficient prognosing of genetic predisposition to urinary bladder carcinoma development.

## Material and methods

The studied group comprised 131 patients with urinary bladder carcinoma, diagnosed for the first time and demonstrating various clinical stages (Ta, T1, T2, T3 and T4) and histological grades of malignancy (G1, G2, G3). DNA from tumour cells and DNA, isolated from peripheral blood, were study materials. The obtained DNA was then submitted to an intensive search for *CHEK2* (IVS2 + 1G>A gene, 1100delC, del5395, I157T) mutation and for polymorphism of *CYP1B1* (355T/T) gene.

In order to find out, whether the searched mutations occurred somatically (being not limited to neoplastic cells) or were constitutional in character, the detection was carried out in the DNA from tumour tissue and from peripheral blood. The assumed presence of oncogenic types of HPV was searched in the DNA isolated from tumour tissue.

The control group included 131 patients (control group I, II), in whom tests were run for identification of *CHEK2* (IVS2 + 1G>A gene, 1100delC, del5395, I157T) mutation and of *CYP1B1* (355T/T) gene polymorphism and oncogenic types of HPV in DNA isolated from epithelial cells in urinary sediment. Seventy-four subjects from control group III were tested for the presence of oncogenic HPV in DNA isolated from epithelial cells in urinary sediment.

## Results

In the study group, a total of 11 mutations of *CHEK2* gene mutations were identified, while 355T/T polymorphism of *CYP1B1* gene was found in 18 cases of the study group (12.9%). In 36 cases (29.3%), out of 123 examined subjects, the presence of an oncogenic HPV type was found. In the control groups (I and II), one I157T missense mutation of *CHEK2* gene was detected. In both control groups, 355T/T polymorphism of *CYP1B1* gene was found in 7 cases.

A study was carried out for the presence of oncogenic types of the virusin 72 subjects of control group II with indication to HPV infection diagnostics, demonstrating the presence of oncogenic HPV in 32 (44.4%) cases. Seventy-four subjects from control group III with no indications to tests for the presence of oncogenic HPV, constituted a reference group. The presence of the virus was identified in 8 (10.81%) cases.

## Conclusions

The performed studies demonstrated a statistically significant difference between the study group and the control group in the incidence of *CHEK2* gene mutations, 355T/T polymorphism of *CYP1B1* gene and the presence of oncogenic HPV types.

Taking into account the obtained results, the following conclusions have been drawn:

1. *CHEK2* gene mutations, 355T/T polymorphism of *CYP1B1* gene and the presence of oncogenic HPV types are observed with a higher, statistically significant prevalence in neoplastic tissue of urinary bladder carcinoma.

2. The concomitance of *CHEK2* gene mutations or 355T/T polymorphism of *CYP1B1* gene and of the presence o oncogenic HPV types statistically significantly correlates with histological malignancy grades of urinary bladder carcinoma.

3. It seems that occurrence of the mutation of *CHEK2* gene, of polymorphism of *CYP1B1* gene and of the oncogenic HPV types can be added to the list of genetic and environmental factors, predisposing to urinary bladder carcinoma development and modifying the course of the disease.

4. Carrying on the above described studies on larger patient populations can allow in the future on the implementation conditions for effective prophylactics: at the carriers of the mutation/polymorphism the elimination/limitation of the influence of the environmental risk factors (occupational and/or the smoking of the tobacco), and early implementation of appropriate antiphlogistic treatment in this antiviral.

